# Dopamine is not essential for the development of methamphetamine-induced neurotoxicity

**DOI:** 10.1111/j.1471-4159.2010.06839.x

**Published:** 2010-08

**Authors:** Jie Yuan, Martin Darvas, Bethany Sotak, George Hatzidimitriou, Una D McCann, Richard D Palmiter, George A Ricaurte

**Affiliations:** * Department of Neurology, Johns Hopkins University School of MedicineBaltimore, Maryland, USA; † Department of Biochemistry and Howard Hughes Medical Institute, University of WashingtonSeattle, Washington, USA; ‡ Department of Psychiatry, Johns Hopkins University School of MedicineBaltimore, Maryland, USA

**Keywords:** dopamine, dopamine-deficient mice, methamphetamine, neurotoxicity, temperature

## Abstract

It is widely believed that dopamine (DA) mediates methamphetamine (METH)-induced toxicity to brain dopaminergic neurons, because drugs that interfere with DA neurotransmission decrease toxicity, whereas drugs that increase DA neurotransmission enhance toxicity. However, temperature effects of drugs that have been used to manipulate brain DA neurotransmission confound interpretation of the data. Here we show that the recently reported ability of l-dihydroxyphenylalanine to reverse the protective effect of alpha-methyl-para-tyrosine on METH-induced DA neurotoxicity is also confounded by drug effects on body temperature. Further, we show that mice genetically engineered to be deficient in brain DA develop METH neurotoxicity, as long as the thermic effects of METH are preserved. In addition, we demonstrate that mice genetically engineered to have unilateral brain DA deficits develop METH-induced dopaminergic deficits that are of comparable magnitude on both sides of the brain. Taken together, these findings demonstrate that DA is not essential for the development of METH-induced dopaminergic neurotoxicity and suggest that mechanisms independent of DA warrant more intense investigation.

The toxic potential of methamphetamine (METH) toward brain dopamine (DA) neurons is well established (Lew *et al.* 1997; Riddle *et al.* 2006; Cadet and Krasnova 2009). Despite considerable research, the precise mechanism(s) by which METH induces degeneration of DA axon terminals remain(s) to be identified. However, a substantial body of data has implicated endogenous brain DA as a mediator of METH neurotoxicity (see Gibb *et al.* 1994; Volz *et al.* 2007; Kuhn *et al.* 2008; for reviews). For example, drugs that deplete brain DA [e.g. alpha-methyl-para-tyrosine (AMPT)] afford neuroprotection (Gibb and Kogan 1979; Schmidt *et al.* 1985), and drugs that replenish DA stores [e.g. l-dihydroxyphenylalanine (l-DOPA)] reinstate METH neurotoxicity (Gibb and Kogan 1979; Thomas *et al.* 2008). These observations have led to that DA mediates METH neurotoxicity (Volz *et al.* 2007; Guillot *et al.* 2008; Thomas *et al.* 2008; Kita *et al.* 2009).

As evidence implicating DA in METH neurotoxicity has accrued, the importance of body temperature in the development and extent of METH neurotoxicity has become apparent (see Sharma *et al.* 2007; Bowyer *et al.* 2008; Krasnova and Cadet 2009). In particular, higher body temperatures enhance toxicity, whereas lower body temperatures generally afford neuroprotection (Bowyer *et al.* 1992; Ali *et al.* 1994; Albers and Sonsalla 1995; Cappon *et al.* 1997; Miller and O’Callaghan 2003). Notably, temperature effects may have direct bearing on studies demonstrating a protective effect of dopaminergic drugs, because most drugs that alter DA neurotransmission also cause alterations in core temperature in METH-treated animals.

We previously reported that near total depletion of brain DA by AMPT or reserpine did not protect animals from METH-induced DA neurotoxicity, as long as drug effects on body temperature were controlled (Yuan *et al.* 2001). Although that study cast doubt on the role of DA in METH neurotoxicity, the experiments involved administration of multiple drugs and complex drug administration paradigms. This left open the possibility that other factors may have been involved in the reinstatement of DA neurotoxicity in DA depleted animals.

The present study sought to more directly evaluate the role of DA in METH neurotoxicity by further taking into account drug effects on body temperature and by using mice genetically engineered to lack DA, on either one or both sides of the brain. DA-deficient (DD) mice are particularly useful for evaluating the role of DA in METH neurotoxicity because they make it possible to circumvent problems associated with complex drug regimens and because animals with unilateral brain DA deficits obviate the need for any DA replenishment. Using these approaches, we now present evidence demonstrating that endogenous DA does not play a role in METH-induced dopaminergic neurotoxicity.

## Materials and methods

### Drugs and chemicals

(+)Methamphetamine (METH) hydrochloride was obtained from the National Institute on Drug Abuse (Rockville, MD, USA). AMPT methyl ester, DA hydrochloride, dihydroxyphenylacetic acid (DOPAC), l-DOPA and benserazide were purchased from the Sigma Chemical Co. (St. Louis, MO, USA). Drug doses were calculated as the free base.

### Animals

For AMPT/L-DOPA studies, we used male albino Swiss-Webster mice (Taconic Farms, Germantown, NY, USA) that were 8 weeks of age and weighed 25–35 g at the beginning of the study. For studies involving genetically induced DA deficiency, we used DD mice generated by gene targeting, as previously described (Zhou and Palmiter 1995); these mice (*Th*−/−; *Dbh*^*Th*/+^) carry two inactive *Tyrosine hydroxylase* (*Th*) alleles, one intact *Dopamine β-hydroxylase* allele (*Dbh*^+^), and one *Dbh* allele with a targeted insertion of the *Th* gene (*Dbh*^*Th*^). DD mice were maintained on a mixed C57BL/6 X 129Sv genetic background and required daily treatment with l-DOPA (50 mg/kg; i.p.) for survival. The METH toxicity experiments were initiated 16 h after the last l-DOPA injection, when mice are > 99% depleted of striatal DA (Kim *et al.* 2000). For studies in animals genetically engineered to lack DA on only one side of the brain, we used dopamine-deficient floxed stop (DDfs) mice that had undergone unilateral viral rescue of dopamine signaling as described (Heusner *et al.* 2003; Hnasko *et al.* 2006; Darvas and Palmiter 2009) using CAV2-Cre virus (Kremer *et al.* 2000). This virus is retrogradely transported from the site of injection and reactivates the endogenous *Th* gene to all DA neurons that project to the injection site, thereby restoring dopamine synthesis and release exclusively in that brain region. The CAV2-Cre virus (0.5 μL at a titer of 6 × 10^12^ particles/mL) was injected into the striatum (0.9 mm anterior to bregma, ± 2.0 mm lateral to midline, 3.0 mm ventral from the skull surface) of anesthetized (isoflurane) 2- to 3-month-old male and female DDfs and control mice that have at least one functional *Th* gene (referred to as sham controls). Virally injected DD mice were removed from l-DOPA treatment 2 weeks after viral injection, and those mice that maintained body weight after 1 week without l-DOPA treatment were designated as virally rescued DDfs (vrDDfs) mice and allowed at least five more weeks of recovery before testing. All mice were housed individually in transparent plastic cages and maintained on a 12 : 12 light-dark cycle (light from 7 am to 7 pm) in a temperature-controlled room (22 ± 1°C), except when otherwise dictated by the experimental design. Food and water were provided *ad libitum*. All animal care and experimental manipulations were approved by the Institutional Animal Care and Use Committees at Johns Hopkins University and the University of Washington, and were in accordance with the National Institutes of Health Guide for the Care and Use of Laboratory Animals. The facilities for housing and care of the animals are accredited by the American Association for the Assessment and Accreditation of Laboratory Animal Care.

### Drug treatments

For AMPT/L-DOPA studies, METH was administered i.p at a dose of 7.5 mg/kg every 2 h for a total of four doses, AMPT was given at a dose of 100 mg/kg, was given i.p. 24, 16, 4 and 1 h before commencing METH treatment, and l-DOPA was given i.p at a dose of 50 mg/kg 1 h before and 3 h after starting METH treatment, always along with benserazide (25 mg/kg; i.p.) to block peripheral l-DOPA decarboxylation. Doses and schedules of drug administration for the AMPT/L-DOPA study were selected on the basis of previous studies in which METH was administered alone and in combination with other drugs (Yuan *et al.* 2001; Thomas *et al.* 2008). For studies involving DD mice, METH was given i.p. at a dose of 10 mg/kg, every 2 h for a total of four doses. The 10 mg/kg dose (instead of 7.5 mg/kg) of METH was selected because it has been used extensively in the past to produce DA lesions (Albers and Sonsalla 1995; see Cadet and Krasnova 2009) and because METH was not used in combination with other drugs in these studies.

### Temperature measurements

Core (rectal) temperature was measured using a Bat-12 thermometer coupled to a RET-3 rectal probe (Physitemp, Inc., Clifton, NJ, USA). Temperature was measured at baseline and every 30 min after METH for up to 4–8 h. In experiments with DD mice that required artificial heating, heat lamps were used, as needed, to ‘yoke’ core temperature in control animals given METH. As before (Yuan *et al.* 2001), this was accomplished by carrying out drug treatments and temperature studies in control mice 1 day prior to carrying out identical studies in DD mice, under identical conditions.

### Chemical and protein determinations

For DA and DOPAC determinations, striatal tissue was dissected 1 week after drug treatment and stored frozen in liquid nitrogen until assay by means of HPLC coupled with electrochemical detection, as described (Villemagne *et al.* 1998). DA transporter (DAT) levels were determined by binding of [^3^H]WIN-35,428, while VMAT was measured by binding of [^3^H]DTBZ as described (Villemagne *et al.* 1998). Tyrosine hydroxylase (TH) was detected in coronal 30-μm brain sections through the mid-brain by using rabbit anti-TH (1 : 2000; Chemicon, Temecula, CA, USA) and CY3-(TH) labeled IgG secondary antibodies (1 : 200; Jackson Immuno-Research, West Grove, PA, USA).

### Glial fibrillary acidic protein immunohistochemistry

Mice were treated with METH (10 mg/kg i.p., every 2 h × 4; *n* = 3) or the vehicle (*n* = 3) and examined 3 days after treatment. Under deep pentobarbital anesthesia (40 mg/kg, i.p.), the animals underwent intracardiac perfusion with 10% formol saline. The brain was then removed from the skull and tissue was sectioned and processed for glial fibrillary acidic protein (GFAP) immunocytochemistry. Tissue blocks were placed in buffered 4% paraformaldehyde for 6 h and then in 10% dimethylsulfoxide in phosphate-buffered saline overnight. Blocks were frozen-sectioned (30 μm) using a sliding microtome and collected in cold phosphate-buffered saline. Free-floating sections were incubated at 4°C for 60 h with rabbit anti-GFAP diluted 1 : 40 in SuperBlock blocking buffer with 0.2% Triton X-100 and 1% normal goat serum. Bound immunoglobulins were visualized with the Vectastain ABC-peroxidase method and staining was enhanced with a standard osmiophilic reaction sequence.

### Data analyses

Temperature results were analyzed by two-way analysis of variance (anova) for repeated measures with treatment as the between subjects factor and time as within subjects factor. When appropriate, group means at individual time points were compared by one-way anova, and *post hoc* comparisons were performed using Duncan’s Multiple Range Test. Striatal DA, DOPAC, DAT and VMAT data were analyzed by one-way anova, followed by Duncan’s Multiple Range *post hoc* comparisons. Correlations were explored using Pearson’s product moment correlation. Results were considered significant when *p* was less than 0.05, using a two-tailed test. Data analyses were performed using a statistical program for the social sciences (SPSS v.16, Chicago, IL, USA).

## Results

**Fig 1:**
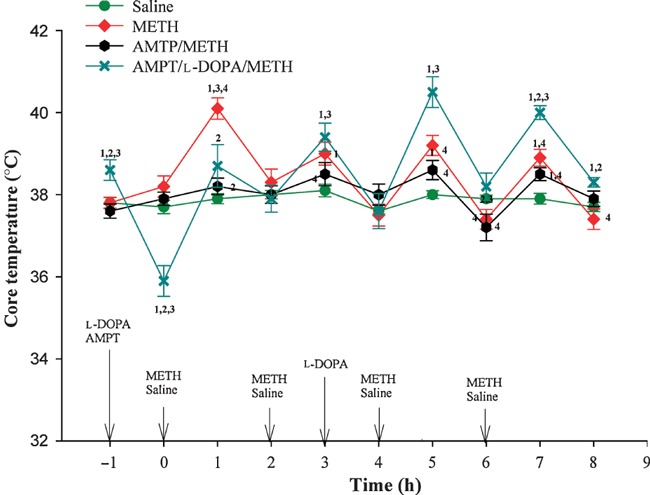
Effect of treatment with METH alone or in combination with AMPT and other drugs on core temperature in mice housed at an ambient temperature of 22 (± 1)°C. For doses and schedules of drug administration, please refer to *Methods*. Values represent the mean ± SEM.; *n* = 6–9 per group. ^1^Designates significant difference from saline control group, ^2^Designates significant difference from METH group, ^3^Designates significant difference from AMPT/METH group and ^4^Designates significant difference from AMPT/L-DOPA/METH group. Significant differences were determined by one-way anova followed by Duncan’s multiple range test. For the sake of clarity, control groups treated with only AMPT, l-DOPA or AMPT + l-DOPA are not shown in the figure.

In keeping with previous reports (Albers and Sonsalla 1995; Kuhn *et al.* 2008), METH injections produced increases in body temperature (Fig. 1). METH-induced increases in body temperature were attenuated by AMPT, particularly at the earlier time points (Fig. 1). l-DOPA increased body temperature in METH-treated animals pre-treated with AMPT (Fig. 1). AMPT alone and l-DOPA alone (or in combination with AMPT) tended to lower core temperature (not shown in figure for the sake of clarity).

**Fig 2:**
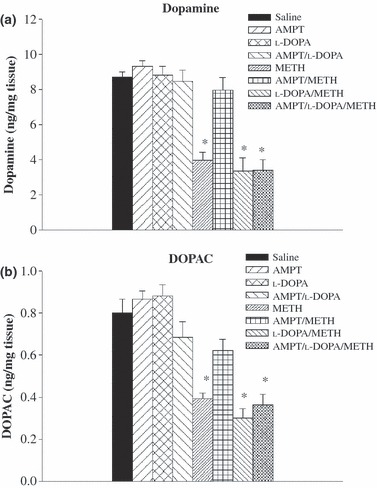
Effect of treatment with METH alone or in combination with other drug treatments on DA and DOPAC concentrations in the striata of mice whose core temperature changes after drug treatment are shown in Fig. 1. DA and DOPAC determinations were performed 1 week after drug treatment. For doses and schedules of drug administration, please see *Methods*. Values represent the mean ± SEM; *n* = 6–9 per group. *Designates significant difference from saline control group. Significant differences were determined by one-way anova followed by Duncan’s multiple range test.

**Fig 3:**
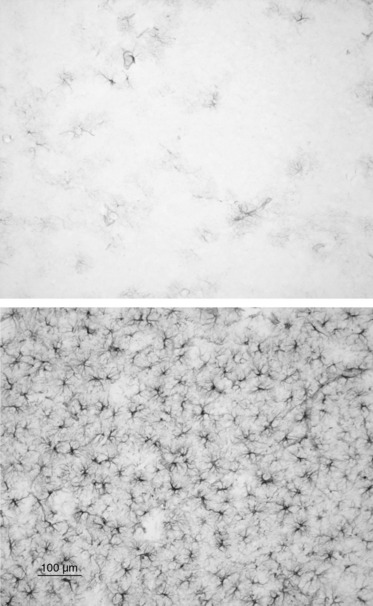
Astroglial activation, as revealed by increased in GFAP staining, in the striatum of DD mouse treated with METH (10 mg/kg, given i.p. every 2 h × 4) 3 days previously (lower panel) compared to control (upper panel).

Striatal DA and DOPAC levels measured 1 week after drug treatment in the various groups of mice described above showed that METH produced significant depletions of striatal DA and DOPAC, and that the effects of METH on DA and DOPAC levels were blocked by AMPT (Fig. 2). l-DOPA reversed the protective effects of AMPT on striatal DA and DOPAC concentrations (Fig. 2). METH also produced increases in GFAP staining (Fig. 3), suggesting that decreases in DA neuronal markers (DA, DOPAC and, as shown below, DAT and VMAT) are secondary to METH-induced dopaminergic neurotoxicity.

**Fig 4:**
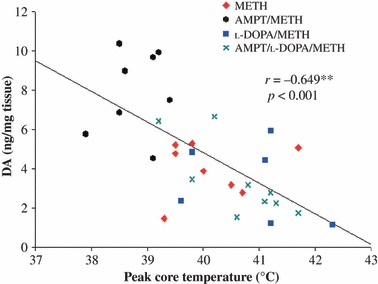
Significant inverse relationship between peak core temperature and striatal DA concentrations 1 week later in all METH-treated animals (i.e. METH alone, METH + AMPT and METH + AMPT + l-DOPA). Correlations were explored using Pearson’s product moment correlation. **p < 0.01.

There was a highly significant inverse relationship between body temperature and DA neurotoxicity in all METH-treated animals (i.e. animals treated with METH alone, METH plus AMPT, and METH plus AMPT plus l-DOPA), with animals achieving the highest core temperatures having the lowest DA levels 1 week after METH treatment, regardless of the drugs used in combination with METH (Fig. 4).

**Fig 5:**
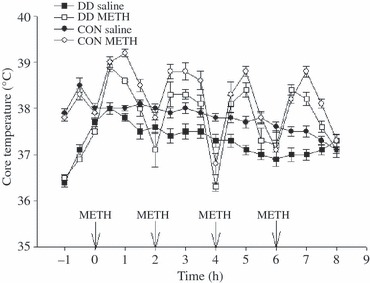
Body temperature responses of mice that received four i.p. injections of saline or METH (10 mg/kg) at 2-h intervals. Arrows indicate time of injection. DD mice were artificially warmed so that their temperature responses to METH paralleled those in WT mice. Values shown are the mean ± SEM; *n* = 5 per group.

**Fig 6:**
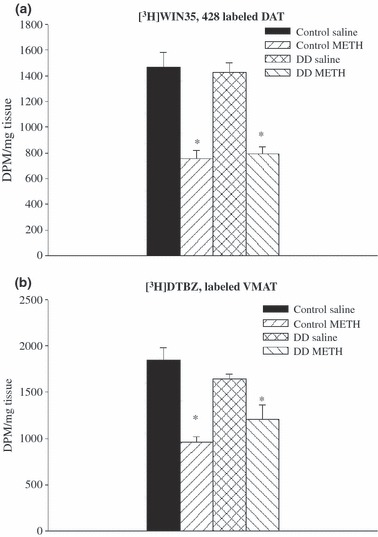
DAT and VMAT densities in the striata of DD and control mice treated 1 week previously with METH or saline. Core temperature responses in these animals are shown in Fig. 4. Values shown are the mean ± SEM; *n* = 5 per group. *Designates significant difference from control group. Significant differences were determined by one-way anova followed by Duncan’s multiple range test.

To further examine the role of DA in METH neurotoxicity, we next evaluated the neurotoxic potential of METH in DD mice that were genetically engineered to have no DA by means of selective inactivation of the *Th* gene within DA neurons (Zhou and Palmiter 1995; Szczypka *et al.* 1999). Given the lack of DA (and, therefore, DOPAC) in these animals, DAT and VMAT served as surrogate DA nerve terminal markers in this experiment. As before, core temperature was monitored before, during and for a period after METH treatment, and DA nerve terminal markers were measured 1 week later. DD mice artificially warmed to develop comparable METH-induced increases in body temperature as those in control mice (Fig. 5) developed significant DAT and VMAT deficits (Fig. 6).

**Fig 7:**
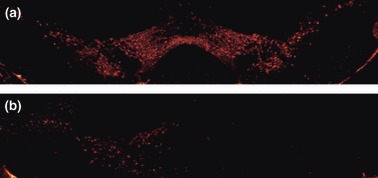
Tyrosine hydroxylase (TH) immunohistochemistry of coronal sections through midbrain of (a) control mouse (b) and a vrDD-DL mouse that had undergone unilateral DA rescue (see *Methods*). DD mice have no TH-positive cell bodies (Hnasko *et al.* 2006).

**Fig 8:**
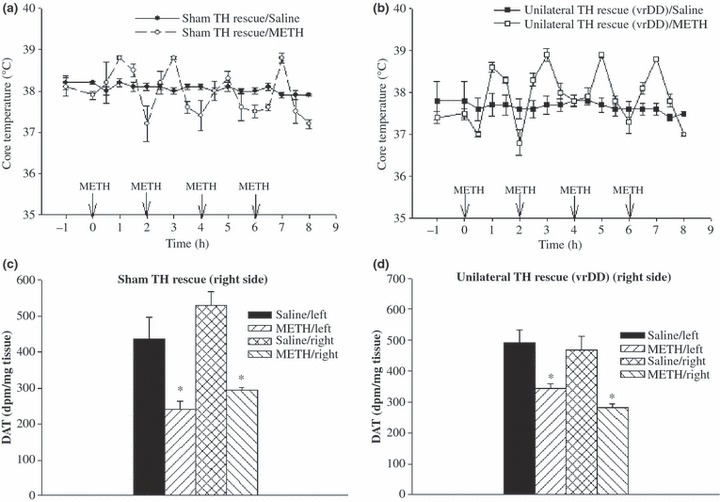
Core temperature responses (top panels) and striatal DAT densities (bottom panels) in unilateral rescue (vrDDL) mice and sham wild-type controls that received four i.p. injections of METH (10 mg/kg) or saline, at 2-h intervals. Core temperature was measured during the period of drug exposure; striatal DAT density was measured 1 week after drug or saline treatment. Given that rescue (or sham procedure) was unilateral and done on right side, right and left striata in each animal were analyzed separately. Note that METH elevates temperature in both sham and virally rescued mice and that the lesions (lasting loss of DAT) are the same on both sides – hence independent of DA. Values shown are the mean ± SEM; *n* = 4 per group. *Designates significant difference from ipsilateral control; significant differences were determined by Student’s *t*-test (*p* < 0.05).

Although previous studies have shown that DA levels in DD mice are < 1% those in wild-type mice 16 h after treatment with l-DOPA (required for survival) (Szczypka *et al.* 1999; Kim *et al.* 2000), there was the remote possibility that a small amount of residual DA might have mediated the effect of METH on DA neuronal markers in DD mice. We, therefore, carried out an additional study in mice in which DA signaling had been restored to only one side of the striatum, DDfs mice (Hnasko *et al.* 2006; Darvas and Palmiter 2009). In contrast to DD mice with bilateral DA deficits, DDfs mice with unilateral DA restoration (Fig. 7) require no daily l-DOPA for survival (Hnasko *et al.* 2006; Darvas and Palmiter 2009). Further, these mice require no warming to develop METH-induced increases in body temperature comparable to those in controls (Fig. 7, *panels A and B*). As shown in Fig. 8*(panels C and D)*, animals with DA restored to only the right striatum developed lasting METH-induced decreases in DAT density that were of comparable magnitude in both striata (right and left).

## Discussion

The importance of DA in METH-induced dopaminergic neurotoxicity has been largely inferred from pharmacological studies demonstrating a protective effect of the catecholamine synthesis inhibitor, AMPT, and a reversal of this effect by treatment with l-DOPA. The first such observation (Gibb and Kogan 1979) was made before the importance of temperature on METH-induced neurotoxicity had been discovered. Therefore, in these early studies in rats, temperature of animals was not measured. Further, DA markers in these studies were measured only 18 h after drug treatment, leaving open the possibility that the effects observed were related to pharmacological rather than toxic drug actions. More recently, Thomas *et al.* (2008) conducted a similar set of studies in mice, and these investigators also reached the conclusion that METH neurotoxicity was mediated by DA (newly synthesized pool). Of note, these recent studies also measured DA axonal markers only 2 days following drug treatment and, therefore, also may have been confounded by pharmacological drug effects. More importantly, as shown in the present study, l-DOPA not only has the potential to replenish DA stores depleted by AMPT but also to raise body temperature in METH-treated animals pre-treated with AMPT (Fig. 2). Thus, the effects of l-DOPA, like those of various other dopaminergic drugs on METH toxicity, are confounded by drug action on body temperature, making it impossible to exclude the possibility that effects of l-DOPA on body temperature, rather than on brain DA levels *per se,* underlie the ability of l-DOPA to reverse the neuroprotective effect of AMPT in METH-treated animals (Gibb and Kogan 1979; Thomas *et al.* 2008).

The important influence of temperature, as opposed to DA, in METH neurotoxicity, is readily apparent in an analysis of the relationship between peak temperature following METH and subsequent neurotoxic lesions in the four groups of METH-treated mice described here. In particular, peak temperature in all METH-treated mice (i.e. animals treated with METH alone, METH + AMPT. METH + l-DOPA and METH + AMPT + l-DOPA) is highly and significantly associated with extent of neurotoxicity (as measured by DA and DOPAC depletion 1 week later), both within and across treatment groups (Fig. 4). For example, animals that received METH + AMPT + l-DOPA and developed higher peak temperatures had greater DA deficits than those in the same group who developed lower peak temperatures. Further, lesion size in the METH + AMPT + l-DOPA group was similar to lesion size in the METH + AMPT group at any particular peak core temperature. These and previous observations (Bowyer *et al.* 1994; Albers and Sonsalla 1995; Yuan *et al.* 2001; Miller and O’Callaghan 2003) strongly suggest that it is the effects of AMPT, l-DOPA and other dopaminergic drugs on core temperature (rather than DA) that influence the expression of dopaminergic neurotoxicity in METH-treated animals. To our knowledge, there is no direct evidence that increased temperature *per se* can cause a lasting decrease of DA axon terminal markers such as DA or DAT, although other neural elements may be adversely affected by hyperthermia (see Kiyatkin 2010).

To further and more directly examine the importance of DA in METH neurotoxicity, we next evaluated the neurotoxic potential of METH in mice deficient in brain DA (DD mice). DD mice artificially warmed to develop comparable METH-induced increases in body temperature as METH-treated controls sustained DAT and VMAT decrements that were of comparable magnitude as those seen in wild-type controls (Fig. 6). This would not be expected if DA was essential for the expression of METH neurotoxicity. That is, if DA was needed for METH neurotoxicity, one would expect at least some decrement in METH-induced toxicity in DD mice, and this was not observed.

Because DD mice can have a small amount of residual DA (< 1% of control), there was the remote possibility that the small amount of remaining DA may have mediated the toxic effect of METH in DD animals. To exclude this possibility, an additional study was carried out using mice genetically engineered to lack DA on only one side of the brain (Hnasko *et al.* 2006; Darvas and Palmiter 2009). Unlike animals with bilateral DA deficits, animals with unilateral DA deficits do not require daily administration of l-DOPA for survival and develop normal temperature elevations after METH, thereby eliminating potential concerns regarding the effects of these factors (l-DOPA, warming) on experimental results. Further, because each animal serves as its own control, issues of inter-animal variability are eliminated. In animals with unilateral brain DA deficits, METH produced lasting bilateral DAT loss that was of comparable magnitude on both sides of the brain, indicating that expression of METH neurotoxicity was not influenced by the presence or absence of DA. Given this finding, it is extremely unlikely that DA is a mediator of METH-induced dopaminergic neurotoxicity.

Potential mechanisms of METH toxicity that are independent of endogenous DA warrant attention. These include: (i) a direct toxic effect of METH (or metabolite) on mitochondrial function leading to energy compromise within DA terminals (Thrash *et al.* 2009); (ii) ionic disturbance within the DA terminals resulting from prolonged interaction between METH and the DAT (Callahan *et al.* 2001); (iii) glutamate-mediated production of free radicals and oxidative stress (Yamamoto and Zhu 1998); (iv) mitochondrial K^+^ ATP channel opening or other molecular events that lead to activation of toxic cellular processes (see Goñi-Allo *et al.* 2008; Cadet and Krasnova 2009); and/or (v) activation of cell death cascades that result in neural injury of the type produced by METH which, notably, can include nerve cell bodies that do not contain DA (see Stirling and Stys 2010). At present, it is not clear which, if any, of these mechanisms is responsible for destruction of DA neuron terminals in METH-treated animals (see Riddle *et al.* 2006; Krasnova and Cadet 2009). However, as detailed elsewhere (McCann and Ricaurte 2004), whatever mechanism is at work must account for the marked influence of body temperature, the key role of the DAT and the sparing of noradrenergic neurons invariably observed in animals that sustain METH-induced DA neural injury.

It is important to acknowledge potential limitations of the present study. First, like previous pharmacological studies, the AMPT/L-DOPA studies described herein involved multiple drugs and complex drug administration paradigms that complicate study interpretation. Second, it is possible that temperature curves in artificially warmed DD animals with bilateral DA deficits did not perfectly model those in control animals, or that the small amount of residual DA in DD animals (< 1%) played a role. However, if DA was essential for METH toxicity, some attenuation of METH toxicity would be expected in DD animals, and this was not observed. Further, DD animals with unilateral DA deficits did not receive l-DOPA or require warming and these animals had similar METH-induced lesions on both sides of the brain, another finding that would not be expected if DA was the mediator of METH neurotoxicity. Finally, there is the outside possibility that DAT and VMAT decreases in METH-treated DD animals are not due primarily to DA terminal destruction but rather reflect METH-induced decreases in the synthesis of these proteins related to actions of METH on the cell nucleus (Asanuma *et al.* 2000). At odds with this possibility is the fact that METH-induced deficits in DA axon terminal markers are associated with signs of glial activation, a finding that is more in keeping with a toxic action of METH on DA axon terminals.

In conclusion, the present findings strongly indicate that endogenous DA is not essential for the development of METH-induced dopaminergic neurotoxicity. Additional research will be needed to identify the series of cellular and subcellular processes that mediate METH-induced DA dopaminergic neurotoxicity. In light of the present findings, neurodegenerative mechanisms independent of endogenous brain DA warrant increased attention.

## Abbreviations

AMPT: alpha-methyl-para-tyrosine
DA: dopamine
DAT: DA transporter
DD: DA-deficient
DDfs: DA-deficient floxed stop
DOPAC: dihydroxyphenylacetic acid
GFAP: glial fibrillary acidic protein
l-DOPA: l-dihydroxyphenylalanine
METH: methamphetamine
TH: tyrosine hydroxylase
VMAT: vesicular monoamine transporter
